# Telemedicine retinopathy of prematurity severity score (TeleROP-SS) versus modified activity score (mROP-ActS) retrospective comparison in SUNDROP cohort

**DOI:** 10.1038/s41598-023-42150-w

**Published:** 2023-09-14

**Authors:** Christine L. Xu, Joel Adu-Brimpong, Henry P. Moshfeghi, Tatiana R. Rosenblatt, Michael D. Yu, Marco H. Ji, Sean K. Wang, Moosa Zaidi, Hashem Ghoraba, Suzanne Michalak, Natalia F. Callaway, Jochen Kumm, Eric Nudleman, Edward H. Wood, Nimesh A. Patel, Andreas Stahl, Domenico Lepore, Darius M. Moshfeghi

**Affiliations:** 1 Department of Ophthalmology, Horngren Family Vitreoretinal Center, Byers Eye Institute, Stanford University School of Medicine, 2452 Watson Ct., Rm 2277, Palo Alto, CA 94303 USA; 2 Carleton College, Northfield, MN USA; 3 Department of Ophthalmology, Massachusetts Eye and Ear Infirmary, Harvard Medical School, Boston, MA USA; 4 Department of Ophthalmology, Jones Eye Institute, University of Arkansas for Medical Sciences, Little Rock, AR USA; 5 Viterbi Family Department of Ophthalmology, Shiley Eye Institute, University of California San Diego, La Jolla, CA USA; 6 Austin Retina Associates, Austin, TX USA; 7 Department of Ophthalmology, Boston Children’s Hospital, Harvard Medical School, Boston, MA USA; 8 Department of Ophthalmology, Bascom Palmer Eye Institute, University of Miami Leonard M. Miller School of Medicine, Miami, FL USA; 9 Department of Ophthalmology, University Medicine Greifswald, Greifswald, Germany; 10 Department of Geriatrics and Neuroscience, Catholic University of the Sacred Heart, A. Gemelli Foundation IRCSS, Rome, Italy

**Keywords:** Retinal diseases, Translational research

## Abstract

Identifying and planning treatment for retinopathy of prematurity (ROP) using telemedicine is becoming increasingly ubiquitous, necessitating a grading system to help caretakers of at-risk infants gauge disease severity. The modified ROP Activity Scale (mROP-ActS) factors zone, stage, and plus disease into its scoring system, addressing the need for assessing ROP’s totality of binocular burden via indirect ophthalmoscopy. However, there is an unmet need for an alternative score which could facilitate ROP identification and gauge disease improvement or deterioration specifically on photographic telemedicine exams. Here, we propose such a system (Telemedicine ROP Severity Score [TeleROP-SS]), which we have compared against the mROP-ActS. In our statistical analysis of 1568 exams, we saw that TeleROP-SS was able to return a score in all instances based on the gradings available from the retrospective SUNDROP cohort, while mROP-ActS obtained a score of 80.8% in right eyes and 81.1% in left eyes. For treatment-warranted ROP (TW-ROP), TeleROP-SS obtained a score of 100% and 95% in the right and left eyes respectively, while mROP-ActS obtained a score of 70% and 63% respectively. The TeleROP-SS score can identify disease improvement or deterioration on telemedicine exams, distinguish timepoints at which treatments can be given, and it has the adaptability to be modified as needed.

## Introduction

Telemedicine for retinopathy of prematurity (ROP) screening has moved through proof of concept, validation in clinical studies, deployment in regional networks, and towards widespread scalability^1–3^. Scalability relies on bridging the knowledge gap between ROP screeners and the neonatal intensive care unit physicians, nursing staff, and family members. This can be accomplished with a scoring system that incorporates the key details and features used by ROP specialists for diagnosis and treatment (such as the International Classification of ROP), while also providing a simpler scoring and risk assessment system that is readily digested by those involved in the infant’s care^4–6^.

One such system is the ROP Activity Score (ROP-ActS) proposed by Smith and colleagues and modified by Pivodic and colleagues (mROP-ActS)^7–9^. The mROP-ActS is based on a construct that assigns a value of 0–22, ranging from incomplete retinal vascularization in any zone (mROP-ActS = 0) to Stage 5 in any zone (mROP-ActS = 22). The mROP-ActS works by grouping feature sets (e.g., Zone and Stage and Plus) into a combination of all possibilities of Zone, Stage, and Plus between Stages 1–3, and then endpoints at Stage 0, 4, and 5 and subsequently assigning a severity label of mild, moderate, or severe (Supplemental Table 1). However, these facets are not weighted proportionately or given a consistent score. The mROP-ActS “bundles” Zone, Stage, and Plus together in such a way that the three categories have interdependent values; for example, the value of Plus drops in each Zone as the Stage increases (Supplemental Table 2). Within a Zone, the Stage score remains constant, but the Plus score varies: for Zone I, Plus is 5, 2, or 0 points; for Zone II, Plus is 8, 6, or 0 points; for Zone III, Plus is 4, 3, or 0 points (Supplemental Table 2). This sort of disparity creates a system that does not adequately reflect anatomic or physiologic disease course. Finally, the mROP-ActS has not been updated to reflect either the classifications of Posterior Zone II in the ICROP 3 revision of 2021^4^, the concept of Pre-Plus disease from the ICROP 2 revision of 2005, or the concept of regression^5^. As a result, 2 major issues exist within the mROP-ActS scoring system in the context of grading telemedicine exams: (1) its output of a value (0–22) instead of a comprehensive score, and (2) its inability to monitor for improvement.

We know that in the United States, 5 of the 6 currently accepted treatment indications include Plus disease. Therefore, Plus disease is important in assessing disease severity and progression. Zone is important because all current treatment recommendations involve either Zone I or II. Finally, stage 3 is important because it drives greater than 50% of the treatment indications. From a treatment perspective, indications 1–6 are equivalent and yet they all have a different score in the mROP-ActS system. An alternative ROP score for use in telemedicine screening would ideally incorporate the features that drive outcomes and intervention in a weighted manner, while also allowing assessment of spontaneous or intervention driven improvement.

The purpose of this paper is to describe a modified ROP scoring system, which we have termed the telemedicine ROP Severity Score (TeleROP-SS), and to compare it against the mROP-ActS in a subset of data from the Stanford University Network for Diagnosis of ROP (SUNDROP)^10^ database to assess correlation between the two scores, ability to return a score in cases responding to treatment, and ability to assess disease directionality. Our hypothesis was that the TeleROP-SS would be able to return a score on more patients than the mROP-ActS due to the TeleROP-SS’s ability to incorporate ICROP metrics such as Posterior Zone II, Regression, and Pre-Plus.

## Methods

This work is approved by the Institutional Review Board, IRB #8752, “Stanford University Network for Diagnosis of Retinopathy of Prematurity (SUNDROP),” certified by the Administrative Panel on Human Subjects in Medical Research. This allows for data analysis, image analysis, and statistical testing. Need of informed consent from all subjects and/or their legal guardian(s) was exempted by IRB with the Administrative Panel on Human Subjects in Medical Research, as stated in IRB #8752. We have adhered to the Declaration of Helsinki and human research standards, and all methods and protocols were carried out in accordance with relevant guidelines and regulations as set by the IRB.

### Database curation

The SUNDROP database includes data from 12 NICUs over ≥ 18 years beginning in 2005^11–13^. We abstracted data from the longest continuously serviced NICU in SUNDROP over a 9-year period. These patients were then evaluated for the following variables:

- Medical record number
- Birth date
- Exam date
- Report date
- Image receipt date
- Estimated Gestational Age (EGA, measured in weeks)
- Post Menstrual Age (PMA, measured in weeks)
- Birth weight
- Daily exam weight
- Images (by eye)
- Zone (by eye)
- Stage (by eye)
- Extent (by eye)
- Plus (by eye)
- Quadrants of Plus (by eye)
- Regression
- Treatment

This data was assessed and correlated with the image database as well as the original reports in order to achieve a curated database.

This dataset was then anonymized and de-identified and placed into a secure HIPAA-compliant online storage folder maintained by Stanford University Information Technology.

### Telemedicine retinopathy of prematurity severity score (TeleROP-SS)

There are currently six treatment indications in the western world for treatment of ROP, which are:

1. Zone 1+
2. Zone 1 or II, Stage 2 or 3, +
3. AROP (complicated by Z I/PzII/II with Plus)
4. Zone I or II, Stage 3, 5 continuous hours, Plus, 4Q
5. Zone 1 or II, Stage 3, 8 interrupted hours, Plus, 4Q
6. Zone I, Stage 3

In evaluating these six indications, it is clear that Zone 1 disease, Stage 3 disease, and Plus disease are prominent drivers of treatment indication. We started with the concept of creating a 0 to 100 points scoring system, and we targeted 55 as the treatment threshold, under which treatment would not be indicated and over which treatment would be indicated. We set aside the top 15 points (i.e. scores 86–100) for adverse outcomes, which are not amenable to treatment intervention for prevention of retinal detachment. We then weighted the numbers such that: (1) any combination of Zone 1, Stage 3, and Plus disease would fall into the treatment range; (2) no combination of Zone 1, Stage 3, and Plus disease would return a score that would fall outside of the treatment range; (3) any combination meeting one of the six treatment criteria above would indicate treatment.

To this end, we created the TeleROP-SS (Table 1), in which Zone I and Plus were assigned 30 points each, whereas Stage 3 was assigned 25 points. In combination with the other weighting, and restricting scoring to Zone I, Posterior Zone II, and Zone II because the currently available imaging systems are unable to reliably and reproducibly image into Zone III, this resulted in a scoring system with an effective minimum score of 15 (Zone II, incomplete, no plus or Pre-Plus). We have created the opportunity for the TeleROP-SS to have further amendments based on changes to the ICROP by allowing the score to potentially go below 15 in the future.

**Table 1:** Points allocation for the telemedicine retinopathy of prematurity severity score.

Zone	Points
I	30
PII	20
II	15

Stage	Points
0	0
1 or reactivation 1	3
2 or reactivation 2	10
3 or reactivation 3	25
REGRESS	10
REGRESS-VEGFI	10
REGRESS-LASER	10

Plus	Points
NONE	0
PRE or reactivation PRE	5
PLUS or reactivation PLUS	30
AROP	85

*AROP* Aggressive ROP. PRE = Pre-Plus. REGRESS = Regression. VEGFI = vascular endothelial growth factor inhibition therapy. LASER = laser retinopexy therapy.

Similarly, we introduced the following severity levels (Table 2):

- Low risk: 0–25
- Moderate risk: 26–39
- High risk: 40–54
- Treatment warranted: 55–85
- Adverse outcome: 86–100

**Table 2:** Severity levels in the telemedicine retinopathy of prematurity severity score.

Severity	Zone	Stage	Plus	ETROP classification	TeleROP-SS
Adverse	ANY	5C	Any	n/a	100
	ANY	5B	Any	n/a	95
	ANY	5A	Any	n/a	90
	ANY	4B	Any	n/a	89
	ANY	4A	Any	n/a	86
Treatment-warranted	I	3	+	Type 1	85
	ANY	AROP	Any	n/a	85
	PII	3	+	n/a	75
	I	2	+	Type 1	70
	II	3	+	Type 1	70
	I	1	+	Type 1	63
	I	3	PRE+	Type 1	60
	I	Incomplete	+	Type 1	60
	PII	2	+	n/a	60
	I	3	–	Type 1	55
	II	2	+	Type 1	55
High risk	PII	1	+	n/a	53
	PII	3	PRE+	n/a	50
	PII	Incomplete	+	n/a	50
	II	1	+	Type 2	48
	I	2	PRE+	n/a	45
	II	3	PRE+	n/a	45
	PII	3	–	n/a	45
	II	3	PRE+	n/a	45
	II	Incomplete	+	n/a	45
	I	2	–	Type 2	40
	II	3	–	Type 2	40
Moderate risk	I	1	PRE+	n/a	38
	I	Incomplete	PRE+	n/a	35
	PII	2	PRE+	n/a	35
	I	1	–	Type 2	33
	I	Incomplete	–	n/a	30
	PII	2	–	n/a	30
	II	2	PRE+	n/a	30
	PII	1	PRE+	n/a	28
Low risk	PII	Incomplete	PRE+	n/a	25
	II	2	–		25
	PII	1	–		23
	II	1	PRE+		23
	II	Incomplete	PRE+++		20
	II	Incomplete	PRE++		20
	PII	Incomplete	–		20
	II	Incomplete	PRE+		20
	II	1	–		18
	II	Incomplete	–		15

TeleROP-SS ROP severity score, *AP-ROP* aggressive posterior ROP.

Please note that Treatment Warranted ROP corresponds to Type 1 Early Treatment ROP (ETROP) with the addition of Aggressive ROP (AROP). High risk ROP corresponds with Type 2 ETROP with the addition of Posterior Zone II.

### Retinopathy of prematurity severity versus activity scoring in SUNDROP database

Each eye at each visit was scored for both the TeleROP-SS and the mROP-ActS. The SUNDROP database includes five images per eye per patient per visit, which are taken on wide-field digital imaging by trained nurses. Scoring was performed in an automated fashion using look-up tables. Then, each score was manually verified by the senior grader (DMM).

### Statistical analysis of the retinopathy of prematurity scoring systems

#### Descriptive analysis and data capture measurement

We conducted descriptive analysis. Simple arithmetic was then applied to determine how often the TeleROP-SS and mROP-ActS returned a score for the SUNDROP dataset for the following outcomes: (1) overall, (2) treated eyes.

#### Retinopathy of prematurity scoring system correlation: severity versus activity

We employed the Spearman Rank Correlation Coefficient to assess correlation between the TeleROP-SS and mROP-ActS on a left eye and right eye basis, for eyes requiring treatment and for eyes that were never treated.

#### Linear mixed effects model analyses

Further, to control for repeated measures (same subjects, same eyes, different time points), we conducted mixed linear effects model for each eye to account for the longitudinal nature of the data and repeated measurements. We utilized a mixed effects linear regression model to account for the subjects potentially having different effects on the measurements and thus our ability to predict outcome (i.e., enabling us to consider both the overall trend and the individual differences between subjects in ROP scores). We specified Random Effects (e.g., subjects) to account for the lack of independent observations with repeated measures. We also specified Random Slopes to allow for the effect of a variable to differ between different levels of a grouping variable (e.g., Stage severity).

## Results

The final curated data abstracted from the single NICU with 9 years of continuous data from the SUNDROP database with known outcomes included 311 unique patients (average estimated gestational age: 28.2 weeks, birthweight 1129 g) with 1568 exams and 3136 eye scorings for each scoring system. 168 of the 311 (54%) unique patients are male.

### Comparison of scores

The overall correlation of TeleROP-SS to mROP-ActS was high for both eyes (r = 0.98, figure not presented), and this effect was independent of overall scoring population versus the subgroups of TW-ROP and untreated patients (Supplemental Figs. 1 and 2). However, importantly, TeleROP-SS reflected subsequent need for treatment for more cases than mROP-ActS. For TW-ROP, overall, there were 39 treatment cases. Of these cases, the TeleROP-SS identified correctly 38 of the 39 cases (97.4% accuracy); the mROP-ActS identified correctly 26 of the 39 cases (66.7% accuracy) (Table 3). Examining each eye separately, the TeleROP-SS identified TW-ROP correctly 100% and 95% of the time in the right and left eye respectively, whereas the mROP-ActS identified TW-ROP correctly 70% and 63% of the time, respectively (Table 3).

**Table 3:** Comparison of accuracy in reflecting treatment warranted ROP (TW-ROP).

	mROP-ActS	TeleROP-SS
Overall	0.67	0.97
Right Eye	0.70	1
Left Eye	0.63	0.95

For TW-ROP (Treatment Warranted ROP), overall, there were 39 treatment cases. Of these cases, the TeleROP-SS (ROP Severity Score) identified correctly 38 of the 39 cases (97.4% accuracy); the mROP-ActS (Modified ROP Activity Score) identified correctly 26 of the 39 cases (66.7% accuracy). Examining each eye separately, the TeleROP-SS identified TW-ROP correctly 100% and 95% of the time in the right and left eye respectively, whereas the mROP-ActS identified TW-ROP correctly 70% and 63% of the time, respectively. The mROP-AcTS failed to identify TW-ROP events when the eyes were classified as Posterior Zone II.

The TeleROP-SS returned a score 100% of the time, while the mROP-ActS returned a score 80.9% of the time (Supplemental Fig. 3). For the mROP-ActS, the (in)ability to return a score was predictable and related to 3 elements: (1) No Pre-Plus category, (2) No Posterior Zone II classification, and (3) No Regression analysis.

We tested 4 linear mixed effects models per eye laterality and selected for the best fit using Akaike Information Criterion (AIC) values (Supplemental Table 3).

## Discussion

Telemedicine using wide-angle digital imaging is increasingly utilized for ROP screening. Interpretation and translation of results in a clinically meaningful manner to non-ROP experts can be facilitated by a simplified scoring system with severity levels, analogous to the Diabetic Retinopathy Severity Scale (DRSS)^14^. In the present study, we demonstrate the rationale for a Telemedicine ROP Severity Scale, and we propose a scoring system with weighted elements that could be used to reflect treatment intervention status and adverse outcomes. TeleROP-SS also correlates clinically relevant and accepted disease severity levels such as adverse outcomes, treatment warranted (i.e., Type 1) and high-risk (i.e., Type 2 ETROP) (Table 2). We assess the TeleROP-SS in a real-world telemedicine database of ROP images (SUNDROP) and patients with known outcomes that have been extensively curated and de-identified. We also compare TeleROP-SS to the mROP-ActS for practicality, correlation, and predictive power. Overall, the TeleROP-SS returned data at a higher percentage and was more accurate in reflecting subsequent treatment as compared to the mROP-ActS. The TeleROP-SS gives more granular data than the mROP-ActS (with the inclusion of Posterior Zone II, Pre-plus disease, and regression). These features may impact our ability to monitor disease, progression, and spontaneous improvement following therapy.

The TeleROP-SS performed well in this analysis. When we compared the TeleROP-SS against the validated mROP-ActS in a retrospective patient population of SUNDROP patients with known outcomes with respect to treatment, intervention, spontaneous regression, and retinal detachment status (or lack thereof), the TeleROP-SS compared favorably to the mROP-ActS. The TeleROP-SS is designed specifically to analyze telemedicine images for acute phase ROP screening. By design, it lacks a designation for Zone III or Maturation at this time, as these are not elements that can be reliably or reproducibly captured on photographic images in preterm infants. It incorporates elements that the mROP-ActS does not, specifically the ICROP recognized features of Posterior Zone II, Pre-Plus disease, and Regression. The mROP-ActS also cannot accurately assess treatment response as there is no element to address regression. While the mROP-ActS may have a role in the totality of the description of ROP, TeleROP-SS has the flexibility needed in a telemedicine screening program.

The properties of the TeleROP-SS make it adaptable for use in identifying disease improvement or deterioration on telemedicine exams. Much like the DRSS, which uses a 10–90 scale, the TeleROP-SS ranges from 15 to 100. Both the TeleROP-SS and the DRSS are bidirectional, thus both improvement and deterioration can be demonstrated by a change in the score. The TeleROP-SS and the DRSS can also identify treatment intervention timepoints (e.g., TW-ROP for TeleROP-SS, high-risk PDR in the DRSS).

In following the umbrella nomenclature agreed upon by the ICROP^6^, we noticed that the mROP-ActS is limited by its bundling of the 3 main elements—Zone, Stage, and Plus. By unbundling the elements from the score, and giving each element its own separate weighting, we can achieve a more granular assessment of the disease status in each eye. Furthermore, unbundling allows for the TeleROP-SS to be modifiable and upgradeable, by design. We are not forced to add a new tier of 3 elements; instead, we can add a separate element to the score. Importantly, the act of unbundling removes the complexity inherent to mROP-ActS, where elements have different impact depending on which other variables they are paired with, which implies some inferred knowledge of their interoperability which has never been established. For example, in the mROP-ActS, the variables of Stage and Plus are non-constant, overlap with each other, and depend on Zone:

- Stage 1: ranges from 1 to 10 points
- Stage 2: ranges from 2 to 12 points
- Stage 3: ranges from 5 to 16 points
- Plus: ranges from 2 to 19 points

This contrasts with the TeleROP-SS, in which every element maintains a constant if weighted score (Table 3). This is more aligned with how we think about disease severity and allows for more consistent application of scoring for disease severity levels.

Campbell and colleagues have demonstrated that a 9-point vascular severity score (VSS) with 3 main tiers (e.g., normal, Pre-Plus, and Plus) is both reproducible by expert graders and validated using deep learning algorithms^11,12,15,16^. We can easily incorporate the 9-point VSS into the Normal/Pre-Plus/Plus paradigm by dividing the scores by 9. Furthermore, the recent Longitudinal Evaluation of ROP Grading (LONG ROP Study) highlighted the concept of tempo as assessed by between-observation variability: better, same, worse^13^. A small tempo score can be appended to TeleROP-SS to assess for Stage changes between ordinal levels that do not constitute a state level change yet represent worsening or improvement.

The strengths of this study are that it is well-conceived and has great comparator group, robust longitudinal database with known patient outcomes, strong statistical analysis and correlation. The major limitation of this study is its retrospective nature. We are applying the scoring system to historical databases. While there is a long history of doing this in scoring systems^8^, this remains a limitation of the study. Moving forward, the TeleROP-SS is being validated prospectively by 12 international pediatric retina ROP specialists in a masked fashion in the LONG ROP Study and the results will be reported when they become available^13^. Another real-world limitation is that images were taken by skilled nurse photographers. For telemedicine to be scalable and established at screening centers beyond the SUNDROP network, this would have to be addressed.

The Telemedicine ROP Severity Score allows for simple documentation of disease status including worsening, improvement, and treatment response, resulting in a score that can be easily interpreted by the non-ophthalmological care team while still providing comprehensive information on disease severity.

### Supplementary Information

Supplementary Information.

## Data Availability

The dataset generated during this study is available on an online repository at https://doi.org/10.5281/zenodo.7453758.
