# Supplementary material for: Telemedicine retinopathy of prematurity severity score (TeleROP-SS) versus modified activity score (mROP-ActS) retrospective comparison in SUNDROP cohort
Source: Sci Rep. 2023 Sep 14;13:15219. doi: 10.1038/s41598-023-42150-w (PMC10502047; doi:10.1038/s41598-023-42150-w)
Supplement: Supplementary file 1 — Supplementary Information. [file 41598_2023_42150_MOESM1_ESM.pdf]

Supplemental Figures:

Supplemental Figure 1. Spearman's Rank Correlation Between ROP Activity Score (mROP-ActS) and ROP Severity Score (TeleROP–SS) by Eye Laterality

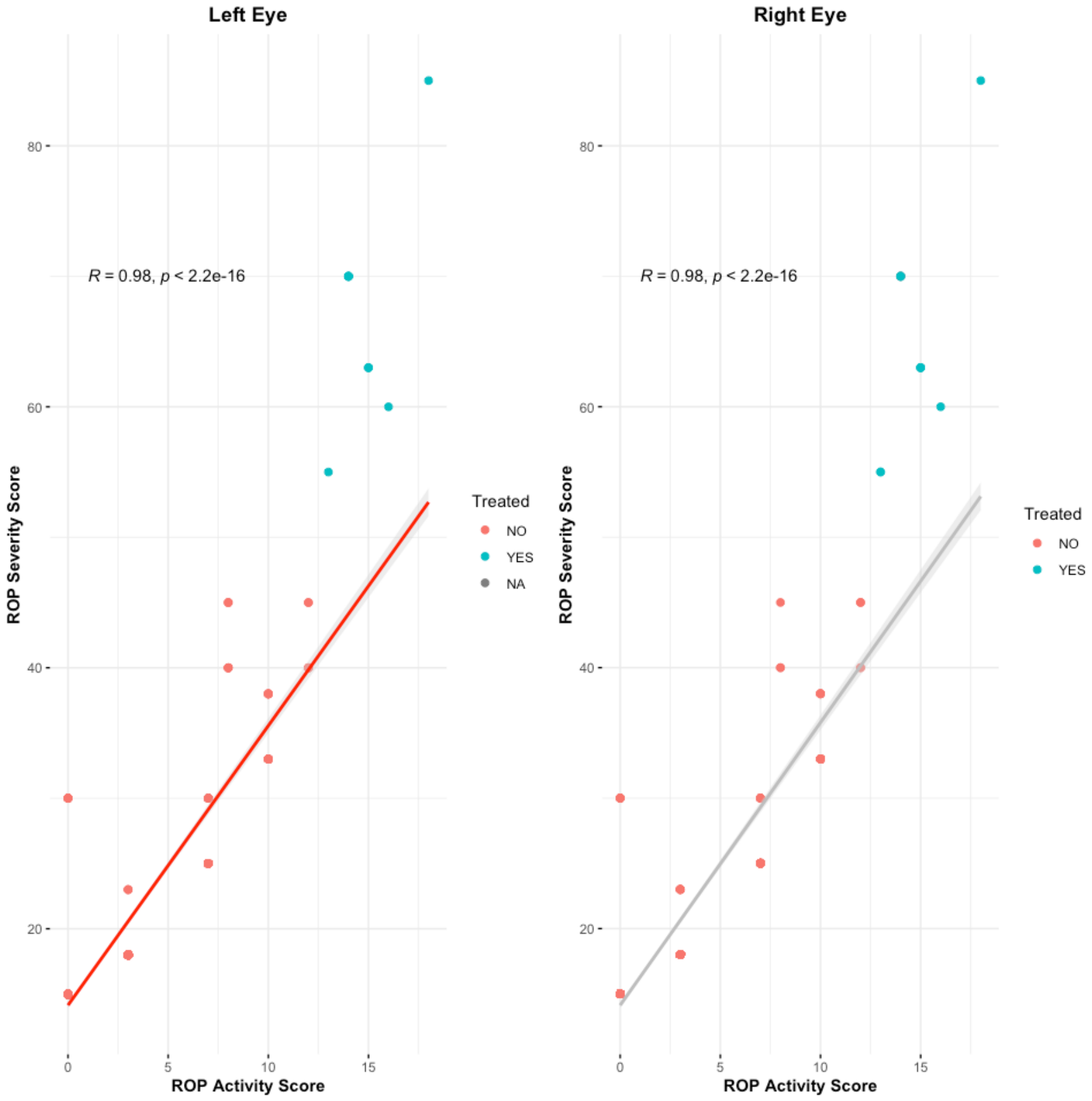

Supplemental Figure 2. Spearman's Rank Correlation Between ROP Activity Score (mROP-ActS) and ROP Severity Score (TeleROP–SS) by Eye Laterality and Treatment

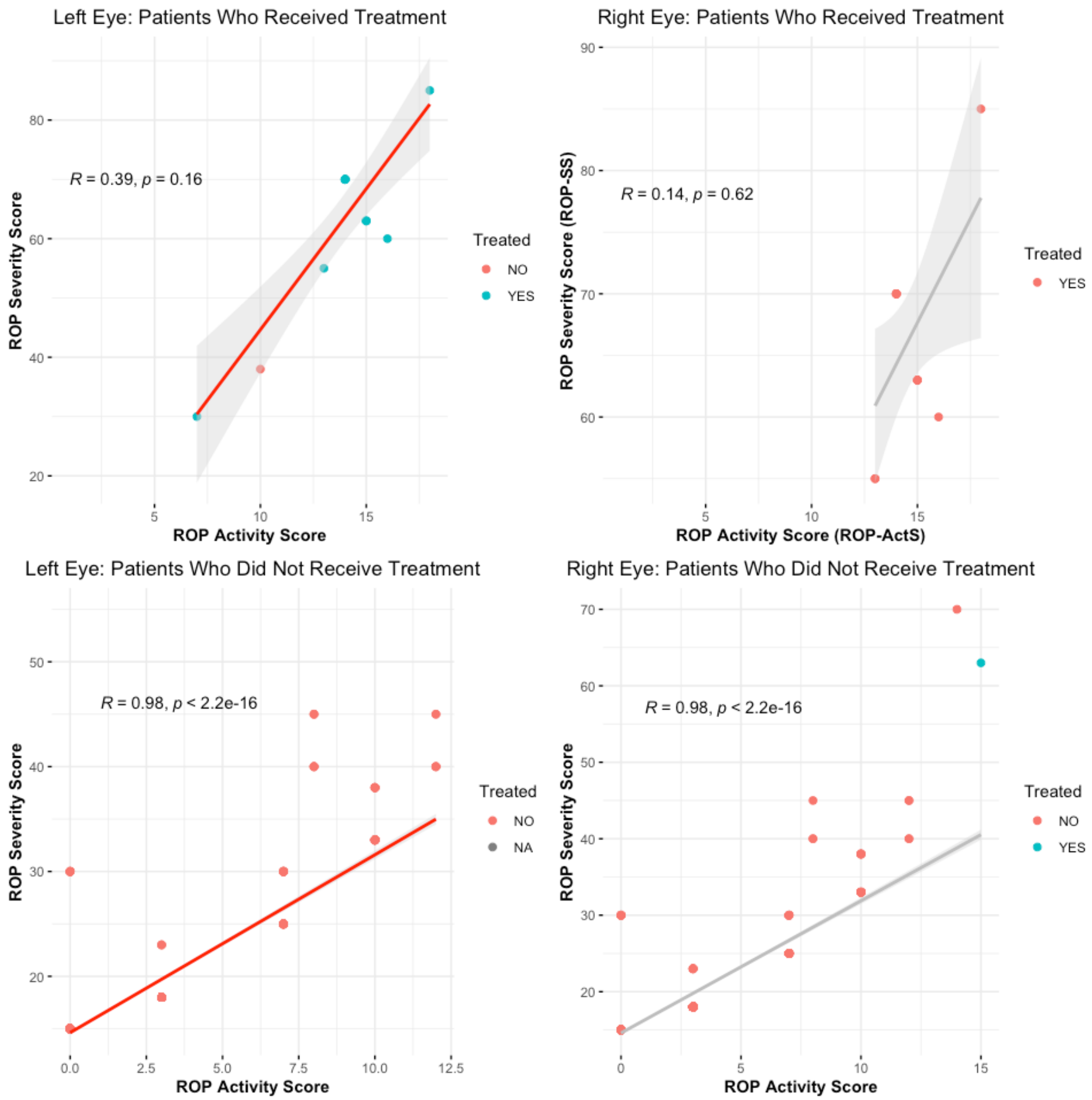

For patients in the "Patients Who Did Not Receive Treatment" category, n=1549. For patients in the "Patients Who Received Treatment" Category, n=20. The low sample size (n) may contribute to the low correlation observed for the "Patients Who Received Treatment" group. Additionally, for the "Left Eye: Patients Who Received Treatment" group, on 1 occasion, both OS_ROPActS and OS_ROPSS methods report scores of 7 and 30, respectively, which still led to treatment even though they did not meet threshold criteria for treatment. Similarly, for the "Right Eye: Patients Who Did Not Receive Treatment" group, there was one individual who met criteria for treatment with OD_ROPActS and OD_ROPSS scores of 14 and 70, respectively who did not receive treatment.

Supplemental Figure 3. Data Capture Rates by ROP Activity Score (mROP-ActS) and ROP Severity Score (TeleROP–SS) in Retinopathy of Prematurity

Overall

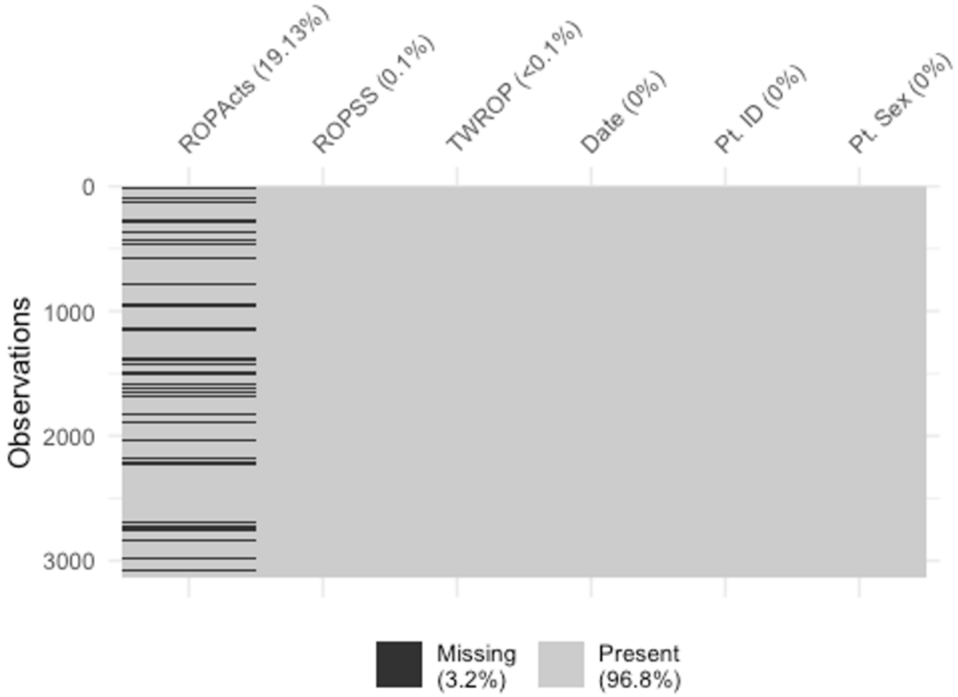

Eye Laterality: Left (OS) vs. Right (OD)

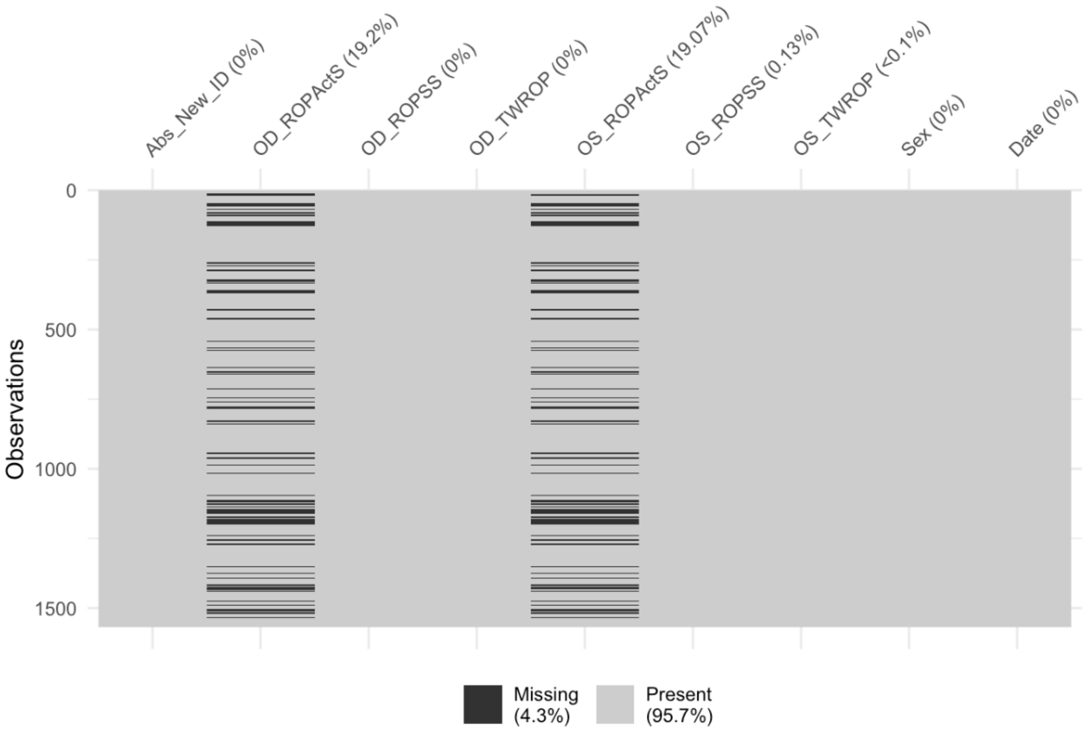

Supplemental Tables:

Supplemental Table 1. Severity Levels in the Modified Retinopathy of Prematurity Activity Scale

ZONE	STAGE	PLUS	SEVERITY	mROP-ActS
ANY	INCOMPLETE	-	mild	0
III	1	-		1
III	2	-		2
II	1	-		5
III	1	+		4
III	3	-		3
III	2	+		6
II	2	-		7
II	3	-	moderate	8
III	3	+		9
I	1	-		10
II	1	+		11
I	2	-		12
II	2	+	severe	13
II	3	+		14
I	1	+		15
I	3	-		16
I	2	+		17
I	3	+		18
ANY	AP-ROP	ANY		19
ANY	4A	ANY		20
ANY	4B	ANY		21
ANY	5	ANY		22

AP-ROP (Aggressive Posterior ROP), mROP-ActS (Modified ROP Activity Score)

Supplemental Table 2. Disparate Values Assigned to Same Features in Retinopathy of Modified Prematurity Activity Score

ZONE	STAGE	PLUS	mROP-ActS	VALUE-PLUS	VALUE-STAGE	STAGE
ANY	INCOMPLETE	-	0	0	0	0
I	1	-	10	0	10	1
I	1	+	15	5	10	1
I	2	-	12	0	12	2
I	2	+	17	5	12	2
I	3	-	16	0	16	3
I	3	+	18	2	16	3
II	1	-	5	0	5	1
II	1	+	11	8	3	1
II	2	-	7	0	7	2
II	2	+	13	6	7	2
II	3	-	8	0	8	3
II	3	+	14	6	8	3
III	1	-	1	0	1	1
III	1	+	4	3	1	1
III	2	-	2	0	2	2
III	2	+	6	4	2	2
III	3	-	3	0	3	3
III	3	+	9	4	5	3
ANY	AP-ROP	ANY	19	19	0	0
ANY	4A	ANY	20	0	20	4
ANY	4B	ANY	21	0	21	4
ANY	5	ANY	22	0	22	5

AP-ROP (Aggressive Posterior ROP), mROP-ActS (Modified ROP Activity Score)

Supplemental Table 3. Mixed Effects Linear Models Predicting mROP-ActS

STATISTIC	RIGHT EYE	LEFT EYE
AIC	3162	3075
F	1382	1719
p-value	<0.0001	<0.001
Interclass correlation coefficient	0.9793	0.9766
Pseudo-R^2 (total)	0.9891	0.9884

We tested 4 linear mixed effects models per eye laterality and selected for the best fit using Akaike Information Criterion (AIC) values. The mixed effects models for the right and left eyes showed that the TeleROP–SS score is a significant predictor of mROP-ActS score.
